# Relationships Between Personal Values and Leadership Behaviors in Basketball Coaches

**DOI:** 10.3389/fpsyg.2018.01661

**Published:** 2018-09-12

**Authors:** Isabel Castillo, Francisco L. Adell, Octavio Alvarez

**Affiliations:** Faculty of Psychology, Universitat de València, Valencia, Spain

**Keywords:** values, behaviors, transformational leadership, perceived pressure, autonomy support, basketball

## Abstract

**Background:** Based on the refined theory of basic individual values and transformational leadership theory, this study focuses on the associations between coaches’ value priorities and their transformational leadership behaviors, exploring the potential mediation versus moderation effect of two alternative variables in this relationship: perceived club pressure or an autonomy supportive environment.

**Methods:** Participants were 266 basketball coaches (85.7% men) from 17 to 66 years old (*M* = 32.82, *SD* = 9.2) from 119 different Spanish clubs. On average, they had worked for their current sport clubs for 5.02 years, and they had a mean of 11.10 years of experience. The coaches were all Spanish speakers, and they trained players at different levels of competition.

**Results:** The stronger the importance of the coaches’ self-transcendent values (i.e., universalism and benevolence), the more they displayed transformational behaviors (i.e., individual consideration, inspirational motivation, intellectual stimulation, and fostering acceptance of group goals) toward the basketball players and perceived a more autonomy supportive environment and lower pressure from the club. Coaches who held conservation values (i.e., humility and face) displayed inspirational motivation behaviors. When coaches held openness to change values (i.e., stimulation and self-direction thought), they tended to display inspirational motivation and intellectual stimulation. Finally, coaches who held beliefs in self-enhancement values (i.e., power) displayed lower transformational behaviors (intellectual stimulation and fostering acceptance of group goals) toward their basketball players, and they perceived higher pressure from the club and a less autonomy supportive environment. Moreover, the club’s autonomy supportive environment played a mediator role between self-transcendence values and some transformational behaviors; however, moderator effects were not significant, with the exception of coaches with self-enhancement values, who tended to avoid intellectual stimulation to a larger extent when they perceived high levels of pressure at the club.

**Conclusion:** These results highlight the importance of identifying the value base on which to develop transformational leadership programs in order to enhance positive experiences in the sport domain.

## Introduction

Values have been defined as desirable goals which serve as guiding principles in people’s life, and influence people’s perceptions, feelings, and behaviors (for a review, see Roccas and Sagiv, 2010), and express different motivational goals (Schwartz, 1992). Values reflect what is important to people, and they are organized in a personal hierarchy of importance. Each person has his/her own hierarchy, and what is important for one person may be not important for another. Values serve as motivators and, according to Schwartz (1992), form a quasi-circular motivational structure. In other words, similar value types are close to each other (such as achievement and power) and appear opposite to conflicting value types (such as conformity and self-direction). Although people differ in how important each value is to them, the structure of motivational congruities and conflicts is nearly universal (Schwartz, 1994; Bardi and Schwartz, 2003). For example, self-transcendence is characteristic of people who are oriented toward the welfare of others, whereas self-enhancement is characteristic of people who are oriented toward self-interest. Conservation is representative of people who want to preserve their status quo, whereas openness to change is representative of people who follow their own intellectual and emotional interests (Schwartz, 1992).

**FIGURE 1:**
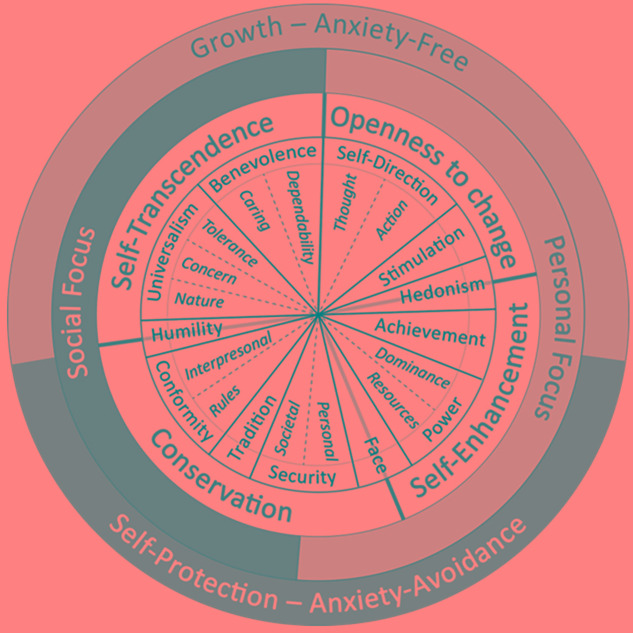
The circular motivational continuum of 19 values in the refined value theory (from Schwartz et al., 2017).

In 1992, Schwartz proposed 10 different values in his original theory of basic human values, and 20 years later, Schwartz et al. (2012) proposed a refined theory identifying 19 conceptually distinct values. These 19 values are sub-dimensions of the 10 basic human values and can be grouped (in both versions) into sets of four higher-order values or dimensions: self-transcendence, self-enhancement, openness to change, and conservation, or even two subsets: growth vs. protection values (see **Figure 1**). Thus, Schwartz et al. (2012) reported that the refined theory gives the option of working with 19 values (i.e., universalism-concern, universalism-nature, and universalism-tolerance; benevolence-dependability and caring; tradition; humility; conformity with rules and interpersonal conformity; personal and societal security; face; power of domination and power over resources; achievement; hedonism; stimulation; self-direction of thought and action). These values cover all of the substantive components of the original 10 values, they combine values and work with the 10 values (i.e., universalism, benevolence, tradition, conformity, security, power, achievement, hedonism, stimulation, and self-direction), or they work with the four higher-order values, organized in two dimensions, that are consistently represented across all human cultures: self-transcendence/self-enhancement and openness to change/conservation. Therefore, depending on the research aims, we can use more broadly or narrowly defined values. Although both versions (classic and refined) describe the same circular motivational continuum, the refined version provides more adjusted predictions and explanations (for definitions of the values, see Schwartz et al., 2012). Consequently, in the present study, this refined version will be used. **Table 1** displays the definition of each value in terms of the motivational goal it expresses.

**Table 1:** The 19 values in the refined theory of values (from Schwartz et al., 2012).

Value dimensions and basic values	Conceptual definitions
**Self-transcendence**	
Universalism-concern	Commitment to equality, justice, and protection for all people
Universalism-nature	Preservation of the natural environment
Universalism-tolerance	Acceptance and understanding of those who are different from oneself
Benevolence-dependability	Being a reliable and trustworthy member of the in-group
Benevolence-caring	Devotion to the welfare of in-group members
**Conservation**	
Tradition	Maintaining and preserving cultural, family, and religious traditions
Humility	Recognizing one’s insignificance in the larger scheme of things
Conformity-rules	Compliance with rules, laws, and formal obligations
Conformity-interpersonal	Avoidance of upsetting or harming other people
Security-personal	Safety in one’s immediate environment
Security-societal	Safety and stability in the wide society
Face	Security and power through maintaining one’s public image and avoiding humiliation
**Self-enhancement**	
Power-dominance	Power through exercising control over people
Power-resources	Power through control of material an social resources
Achievement	Success according to social standards
**Openness to change**	
Hedonism	Pleasure and sensuous gratification
Stimulation	Excitement, novelty, and change
Self-direction-thought	Freedom to cultivate one’s own ideas and abilities
Self-direction-action	Freedom to determine one’s own actions

*Value dimensions are in bold*.

An important reason for studying values is the assumption that values have an important influence on behaviors and can be used to explain and predict behaviors (Roccas and Sagiv, 2010). As Schwartz (2006) proposed, people try to express their important values through their behavior in order to affirm the values that are central to their self-identities and attain the goals that are important to them. Schwartz (2017) pointed out that a value has an influence on behavior when this value has previously been activated. In addition, important values are activated more often and have a special influence on behaviors that serve to promote or inhibit attainment of the goals associated with these values.

Considerable evidence suggests that values have a small but consistent association with a wide variety of behaviors, such as creative behaviors (Kasof et al., 2007), consumer choices (Doran, 2009), and pro-environmental behaviors (Schultz and Zelezny, 1998). Using self-reports related to values and behaviors with a Russian sample, Schwartz and Butenko (2014) confirmed that most of the values positively correlated more with the behavior chosen as likely to express it than with any other behavior, whereas the opposite was true for the values and behaviors that expressed motivationally opposed values that were negatively correlated. Recently, Schwartz et al. (2017) confirmed in four countries (Italy, Poland, Russia, and the United States) that behavior depends on trade-offs between values that promote the behavior and values that inhibit it. More specifically, they suggested that, although several values can be associated with a single behavior, some behaviors are more likely to be motivated predominantly by one value and inhibited by its conceptually opposed values, and these behaviors are called value-expressive behaviors (Bardi and Schwartz, 2003).

In the sport context, as in other achievement domains (e.g., academic context), leaders have an influential and essential role in promoting important cognitive, psychological, and behavioral outcomes (for a review in youth sport and physical education see Whitehead et al., 2013). Specifically in the sport domain, coaches’ behaviors and athletes’ reactions to these behaviors are critical in establishing a high quality coach-athlete relationship (Vella et al., 2010). A large amount of previous research has examined the important effect of coaching behaviors on athletes’ self-determination, effort, sport performance, intention to persist in sport, well-being, burnout, etc. (e.g., Hollembeak and Amorose, 2005; González et al., 2016).

More specifically, research has found that transformational coaches’ behaviors are associated with athletes’ performance outcomes (e.g., Álvarez et al., 2013; Morgan et al., 2015), teams’ task cohesion (e.g., Price and Weiss, 2013), perceived competence, enjoyment, and collective efficacy (e.g., Price and Weiss, 2013), citizenship behaviors (Lee et al., 2013), and athletes’ well-being (Stenling and Tafvelin, 2014). As Álvarez et al. (2016) stated, transformational leadership behaviors are seen as desirable, associated with higher coach efficacy, and highly recommended in order to obtain benefits for athletes (for more details see Álvarez et al., 2016).

The transformational leadership theory (Bass, 1985) has been successfully applied to the sport context, with growing interest since the early 2000s (Álvarez et al., 2016). Transformational leadership is multidimensional in nature (Podsakoff et al., 1990). Transformational leadership refers to the leader moving the follower beyond immediate self-interest through individualized consideration, inspiration, intellectual stimulation, acceptance of group goals, high expectations, and an appropriate role model. In other words, through transformational behaviors, coaches treat each athlete as a prized team member, recognizing individual differences with a supportive leadership style (*individualized consideration*). Coaches motivate and inspire athletes by connecting athletes’ potential to the vision of future group/team states, and by giving them meaning and challenges in everyday activities (*inspirational motivation*). Coaches also stimulate athletes to think in different ways when facing new and old challenges and issues (*intellectual stimulation*) (Bass and Riggio, 2006; Álvarez et al., 2010). A transformational coach promotes cooperation among athletes, getting them to work together toward a common goal (*fosters acceptance of group goals and team work*). Coaches set high expectations for athletes’ behavior and performance (high performance expectations), and they also provide a positive behavioral model for athletes to follow (*provides an appropriate role model*) (Podsakoff et al., 1990; Hardy et al., 2010; Vella et al., 2012). In sum, leaders who display transformational behaviors transform or change the basic values, beliefs, and attitudes of their followers (Podsakoff et al., 1990), acting in accordance with their own personal values and convictions. However, very few empirical studies have investigated the relationship between values and transformational leadership (Groves and LaRocca, 2011).

To date, transformational leadership research has focused on investigating its main effects (see review by Judge and Piccolo, 2004) on the conditions where these leadership behaviors are more or less effective in a variety of settings (e.g., Dvir and Shamir, 2003; Walumbwa and Lawler, 2003; Lim and Ployhart, 2004), and on its relationship with a number of psychological outcomes in athletes (for a review, see Álvarez et al., 2016). However, few studies have explored how aspects of coaches’ social environments affect their behaviors, that is, the role of personal (i.e., values priorities) and environmental conditions in leading coaches to adopt transformational behaviors in their interactions with athletes and team building. Consequently, research is needed on the factors that influence coaches’ use of transformational behaviors.

Given that values seem to play an essential role in understanding leadership (e.g., Sosik, 2005), some researchers have suggested that transformational leaders may be more or less effective depending on societal values (Scandura and Dorfman, 2004), and a number of studies have explored in what way group values influence followers’ responses to transformational leadership, and in what way these responses influence group performance (Schaubroeck et al., 2007), or which follower values are linked to leader preferences (Ehrhart and Klein, 2001). However, in general and only in the organizational context, a limited amount of empirical research has examined how Schwartz’s personal values are related to transformational behaviors (e.g., Sarros and Santora, 2001; Sosik, 2005; Singh and Krishnan, 2014). Sarros and Santora (2001) examined the relationships between value orientations of executives and their transformational behaviors. They found a strong positive association between transformational leadership behaviors and self-transcendence (i.e., benevolence) and openness to change values (i.e., self-direction and stimulation). In addition, in a sample of managers, Singh and Krishnan (2014) found a positive and significant relationship between self-transcendence values and transformational leadership. In the same domain, Sosik (2005) studied how managers’ personal values were related to what is called charismatic leadership, composed of two of the transformational behaviors (i.e., idealized influence and inspirational motivation). Results indicated that traditional values (i.e., honoring parents and elders, and showing respect), self-transcendence, and self-enhancement were positively related to charismatic leadership.

Overall, it seems that the dimension of self-transcendence is consistently related to transformational behaviors, whereas the other dimensions are inconsistent. In model Schwartz (1992), self-transcendence and openness to change are compatible value types, as are self-enhancement and conservation values; whereas these two dimensions are conflicting each other, that is self-transcendence and self-enhancement are motivationally incompatible values as are conservation and openness to change values (see **Figure 1**). Therefore, we can hypothesize that self-transcendence and openness to change values are positively related to transformational behaviors, whereas openness to change and conservation are negatively related or not significantly related to transformational behaviors.

To the best of our knowledge, no publication in the sport context has explored the relationships between Schwartz’s values and transformational behaviors. Accordingly, we aim to examine the relationships between coaches’ value priorities and transformational leadership behaviors in a sample of basketball coaches.

As Bardi and Schwartz (2003) pointed out, the relationship between values and behaviors is partly obscured by norms. Sometimes highly endorsed values and behaviors show weaker value-behavior relationships because normative pressures may induce individuals to comply with group expectations rather than basing their behaviors on their own values. That is, values can influence behavior more when situational pressures are weak. By contrast, values can be opposite to behaviors, when people want to conform to the group especially under heavy pressure rules. In the same vein, strong and weak situations are well-studied and described in personality psychology (cf. Mischel, 1977). A strong situation would be one in which high pressure is exerted so that everyone is forced to behave in a more or less similar way. While in a weak situation people can usually be what they really are because there are few or no restrictions on behavior (for more information on the analysis of person-situation interplay and how to assess situation strength/weakness see Leising and Müller-Plath, 2009). Recently, Schwartz et al. (2017) suggested studying other variables that may moderate the strength of value-behavior relations, for example, other reported behaviors or mechanisms through which values may influence behavior. We examine the generalizability of the normative explanation by examining the role of the perceptions of autonomy supportive or pressure environments at the club in the strength of the value-behavior relations in a sample of basketball coaches.

Self-determination theory (SDT; Deci and Ryan, 2000) suggests that contextual factors affect whether leaders use certain behaviors that transform subordinates or behaviors that pressure subordinates. For example, in the physical education (PE) domain, PE teachers indicated that perceived school administrative pressure to teach in a specific way changed their use of motivational strategies and caused them to display more controlling strategies (Taylor and Ntoumanis, 2007).

**FIGURE 2:**
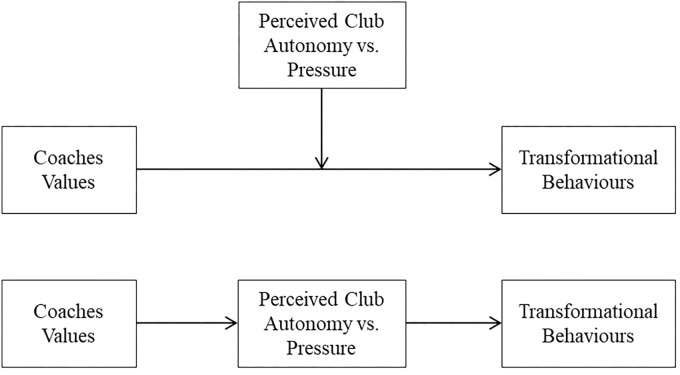
Study model: Relationship between coach values and transformational behaviors, mediated vs. moderated by perception of autonomy vs. pressure at the club.

Based on Schwartz’s refined theory of basic individual values (Schwartz et al., 2012) and transformational leadership theory (Bass, 1985), this study focuses on the associations between coaches’ value priorities and their transformational leadership behaviors, exploring the potential mediation versus moderation effect in this relationship of two alternative variables: the perceived club pressure or autonomy supportive environment at the club (see **Figure 2**). We consider that coaches for whom particular values are especially important may tend to display more transformational behaviors than coaches guided by a different set of values. Transformational behaviors (e.g., individual consideration) may be more strongly associated with emphasizing certain values (e.g., universalism), rather than others (e.g., power) that may not have any significant relationship with transformational behaviors or may even have a negative relationship. We hypothesize that (1) the stronger the intensity of the coach’s self-transcendent values and openness to change values, the more likely the coach is to display transformational behaviors and perceive a more autonomy supportive environment and less pressure from the club. However, (2) coaches who hold self-enhancement and conservation values will display lower transformational behaviors and perceive higher pressure from the club and a less autonomy supportive environment.

## Materials and Methods

### Participants

Two-hundred and sixty-six basketball coaches (85.7% men) ranging from 17 to 66 years old (*M* = 32.82, *SD* = 9.2) from 119 different clubs participated in this study. On average, coaches had worked for their current sport club for 5.02 years, and they had a mean of 11.10 years of experience. The coaches were all Spanish speakers, and they trained players at different levels of competition.

### Design and Procedure

A cross-sectional study was used to examine the associations between coaches’ value priorities and their transformational leadership behaviors, exploring the potential mediation versus moderation effect of two alternative variables in this relationship: the perceived club pressure or the autonomy supportive environment.

This study was conducted in accordance with international ethical guidelines, which are consistent with American Psychological Association guidelines. Ethical approval to conduct the research was obtained from the committee on research ethics of the University of Valencia (Spain) (Reference H1523110229495). Participants were recruited during a basketball practice session held within the 1st month of the basketball season. The coaches were informed that the purpose of the study was to explore basketball coaches’ values. Those wishing to participate were invited to provide written informed consent and to complete an anonymous questionnaire containing instruments in the order listed below, followed by a set of background variables, such as gender, age, level of education, and answer all the questions as accurately and honestly as possible because there were no wrong or right answers. The data were collected between May and September 2017.

### Measures

#### Coach Values

Values were measured using the Spanish version of the 57-item Portrait Values Questionnaire - Refined (PVQ-R; Schwartz et al., 2012), by asking coaches to indicate how similar they are to gender-matched individuals who are described in terms of their important values. The questionnaire starts with the stem, “How much is this person like you?”, and responses ranged from 1 (*not like me at all*) to 6 (*very much like me*). An example item is: “It is important to him to have a good time.” The refined theory based model posits 19 value factors (i.e., universalism-concern, universalism-nature, and universalism-tolerance; benevolence-dependability and caring; tradition; humility; conformity with rules and interpersonal conformity; personal and societal security; face; power of domination and power over resources; achievement; hedonism; stimulation; self-direction of thought and action), each measured with three marker items. Evidence for the reliability and predictive validity of this refined instrument has been shown previously (e.g., Schwartz et al., 2012; Torres et al., 2016). Reliabilities (Cronbach’s alphas) of this instrument and the following can be found in **Table 3**.

#### Perceived Club Autonomy

Coaches’ perceptions of the degree of autonomy support provided by the club were assessed by adapting the seven-item Spanish version (Balaguer et al., 2009) of the Sport Climate Questionnaire^1^ for this study. Each item starts with the phrase: “In this basketball club…”, and the responses are rated on a 7-point scale, ranging from 1 (*does not correspond at all*) to 7 (*corresponds completely*). An example item is “I feel I am provided with choices and options regarding my coaching.” The reliability of this instrument has been shown in previous sport-based studies (e.g., Adie et al., 2008; Balaguer et al., 2012).

#### Perceived Club Pressure

Five items from the Constraints at Work scale (Pelletier et al., 2002), adapted to the sport context, were used to measure coaches’ perceptions of pressure at the club. These items are designed to measure coaches’ perceptions of pressure associated with other colleagues at the club (e.g., The other coaches at my club do not allow me to decide how I coach) and coaches’ perceptions of pressure coming from the club staff (e.g., I wish I could coach in certain ways, but my club doesn’t allow it). The coaches have to answer each item on a 7-point scale, ranging from 1 (*does not correspond at all*) to 7 (*corresponds completely*). The reliability of this scale has been shown in previous studies in the PE context (Pelletier et al., 2002).

#### Coach Behaviors

Coach leadership behaviors were measured using the Differentiated Transformational Leadership Inventory (Callow et al., 2009), adapted to the sport context (Vella et al., 2012) and translated to Spanish for this study. The instrument captures six transformational leadership behaviors, including individual consideration, inspirational motivation, intellectual stimulation, fostering acceptance of group goals and promoting teamwork, high performance expectations, and appropriate role model (each subscale contains four items, except for fostering acceptance of group goals, which contains three items). The 23-item instrument begins with the stem, “As a coach, when I train…”. An example item is: “Treat each team member as an individual” for individual consideration, or “Encourage athletes to be team players” for fostering acceptance of group goals and promoting teamwork. A 5-point Likert scale was used, ranging from 1 (*not at all*) to 5 (*all of the time*). Evidence for the reliability and validity has been provided in previous research conducted in the sport context (e.g., Callow et al., 2009; Vella et al., 2012).

### Data Analysis

The factor structure of all the measures was tested using confirmatory factor analysis (CFA) with LISREL 8.80. We estimated parameters using the maximum likelihood estimator. The following fit indices were examined: Satorra–Bentler scaled chi square (χ^2^); Root Mean Square Error of Approximation (RMSEA); Standardized Root Mean Square Residual (SRMR); Comparative Fit Index (CFI); and Non-Normed Fit Index (NNFI). We considered RMSEA values ≤ 0.08, SRMR ≤ 0.06, CFI and NNFI values ≥ 0.90, to indicate a reasonable model fit.

Following the procedure proposed by Cieciuch and Schwartz (2012), four different CFA were carried out for each of the four types of categories of values, in order to evaluate the degree of distinction of the 19 values and their fit indices. Self-transcendence is composed of the values of universalism-tolerance, universalism-nature, universalism-concern, benevolence-dependence, and benevolence-care. Conservation is composed of the values of tradition, humility, conformity with rules, interpersonal conformity, personal safety, social safety, and face. Self-enhancement is composed of the values of power of domination, power over resources, and achievement. Finally, Openness to Change is composed of the values of hedonism, stimulation, and self-direction of thought and action. To test the underlying factor structure of the DTLI, a six-factor structure was conducted considering the six subscales or six transformational coach behaviors established by Vella et al. (2012), that is, individual consideration, inspirational motivation, intellectual stimulation, fostering acceptance of group goals, high performance expectations, and appropriate role model. Finally, a unidimensional factorial structure was established for the perceptions of the club autonomy support factor and another one for the perceptions of club pressure factor.

We used Hayes (2013) PROCESS macro to determine whether the association between coaches’ value dimensions and their leadership behaviors was moderated (Model 2) or mediated (Model 4) by the coaches’ perceptions of pressure and autonomy support provided by the club (see **Figure 2**). We used the bootstrapping method based on 1000 samples to estimate standard errors of indirect effects.

The IBM SPSS statistics 20 was used to analyze the descriptive statistics and the homogeneity indexes (corrected item-total correlations), as well as the Pearson correlations between the study variables. Following Schwartz et al. (2012), we used value priorities when computing descriptive statistics (means, standard deviations, and correlations), based on the relative importance of each value to each person derived by centring each person’s responses on his/her own mean. By doing that we control the individual response tendencies as well as the interrelationship of the values within the circular structure, reflecting the expected compensation of opposing values. The results conducted on raw value scores can be found in the **Supplementary Material** (**Supplementary Tables S4B**, **S5B**). We also used centered responses for regression analyses because we introduced one value dimension each time as predictor variable. We used not centered responses in all other analyses (CFA and internal consistency coefficients).

## Results

The CFA results revealed that the proposed factorial structure was acceptable for each study instrument (see **Table 2**). All the scales were found to have an acceptable model fit, and all the standardized factor loadings for each item in its designated factor were greater than 0.40, *p* < 0.001, which also indicated that no items should be dropped. For reasons of brevity, the results of the CFAs are not presented here, but they are available in the **Supplementary Material**.

**Table 2:** Confirmatory factor analyses: goodness of fit indices for all study instruments.

Models	χ^2^	*df*	RMSEA	(90% CI)	SRMR	NNFI	CFI
Self-transcendence	217.64	80	0.080	(0.068–0.093)	0.048	0.948	0.961
Conservation	363.01	168	0.066	(0.056–0.075)	0.054	0.935	0.948
Self-enhancement	94.09	24	0.083	(0.063–0.089)	0.066	0.921	0.939
Openness to change	132.45	48	0.081	(0.065–0.087)	0.053	0.944	0.960
Transformational leadership	311.61	215	0.075	(0.066–0.083)	0.068	0.910	0.920
Perceived club autonomy support	39.03	14	0.047	(0.039–0.070)	0.049	0.946	0.964
Perceived club pressure	5.09	5	0.052	(0.030–0.067)	0.040	0.952	0.958

*For all χ^2^ values, p < 0.001; df, degrees of freedom; RMSEA, root mean square error of approximation; 90% CI, 90% confidence interval for the RMSEA; SRMR, standardized root mean residual; NNFI, non-normed fit index; CFI, comparative fit index*.

Means, standard deviations, skewness, kurtosis, range, and Cronbach alpha coefficients are displayed in **Table 3**. An inspection of the mean-centered value scores shows that coaches reported that self-transcendence and openness to change represented their value priorities, and that conservation and self-enhancement were the least important value dimension for their priorities. Coaches perceived that they displayed high levels of transformational behaviors, they reported low levels of pressure at the club, and they reported a high autonomy supportive environment provided by the club. Total scales and subscales showed acceptable (alpha > 0.70) or marginally acceptable (alpha > 0.60) internal consistency, except for four basic values (humility, achievement, self-direction-thought, and self-direction-action) and two transformational behaviors (individual consideration and appropriate role model), which were unacceptable (alpha < 0.60). Nevertheless, none of the items would have substantially increased the alpha coefficient if deleted, and so we retained these scales because the factor loadings for the observed indicators were satisfactory. Results related to these subscales should be interpreted with caution and need to be confirmed in other studies.

**Table 3:** Descriptive statistics and reliability of all the study variables (19 basic values, 4 value dimensions, coach behaviors, perceived club autonomy and perceived club pressure) (*N* = 266 coaches).

	Range	Mean	*SD*	Alpha	Skewness	Kurtosis
Universalism-concern	1–6	5.16	0.64	0.74	-0.46	0.12
Universalism-nature	1–6	4.46	0.89	0.86	-0.52	-0.18
Universalism-tolerance	1–6	5.06	0.66	0.66	-0.52	0.83
Benevolence-dependability	1–6	5.39	0.49	0.70	-0.56	0.77
Benevolence-caring	1–6	5.37	0.55	0.65	-0.64	0.52
Tradition	1–6	3.26	1.02	0.78	-0.10	-0.60
Humility	1–6	4.49	0.78	0.40	-0.54	0.88
Conformity-rules	1–6	4.36	0.84	0.80	-0.73	0.74
Conformity-interpersonal	1–6	4.38	0.90	0.76	-0.50	0.23
Security-personal	1–6	4.62	0.60	0.60	-0.74	1.00
Security-societal	1–6	4.31	0.88	0.74	-0.37	0.01
Face	1–6	4.27	0.84	0.66	-0.47	-0.11
Power-dominance	1–6	2.84	0.96	0.74	0.28	-0.03
Power-resources	1–6	2.33	0.93	0.72	0.40	-0.13
Achievement	1–6	4.27	0.72	0.54	-0.12	0.05
Hedonism	1–6	5.20	0.61	0.70	-0.17	-0.22
Stimulation	1–6	4.37	0.86	0.77	-0.30	0.35
Self-direction-thought	1–6	5.13	0.62	0.56	-0.00	-0.25
Self-direction-action	1–6	5.04	0.65	0.53	-0.40	-0.12
Self-transcendence	1–6	5.09	0.35	0.85	-0.17	0.07
Conservation	1–6	4.24	0.38	0.85	-0.42	0.45
Self-enhancement	1–6	3.14	0.65	0.78	0.03	-0.10
Openness to change	1–6	4.94	0.46	0.81	0.12	0.00
Individual consideration	1–5	4.28	0.50	0.47	-0.34	-0.66
Inspirational motivation	1–5	4.23	0.56	0.72	-0.49	-0.28
Intellectual stimulation	1–5	4.11	0.51	0.69	-0.54	0.60
Fostering acceptance group goals	1–5	4.49	0.53	0.69	-1.01	0.45
High performance expectations	1–5	4.21	0.55	0.60	-0.55	-0.35
Appropriate role model	1–5	4.07	0.47	0.47	-0.23	-0.20
Perceived club autonomy	1–7	5.13	1.11	0.82	-0.60	0.00
Perceived club pressure	1–7	2.01	0.90	0.74	1.27	1.72

*Means and SD for values reflect value priorities and are based on centring each coach’s responses around his/her mean for all 57 items and then adding the overall mean (4.4363) for all respondents to the same scale to restore the original scale. Alphas were computed using not centered responses*.

As expected, positive – albeit weak-, correlations were found between basic values and transformational coach behaviors (see **Table 4**). Universalism-concern and universalism-tolerance were positively related to individual consideration, intellectual stimulation, and fostering acceptance of group goals behaviors, whereas universalism-nature was only positively related to intellectual stimulation. Benevolence-dependability and benevolence-caring were both positively associated with fostering acceptance of group goals. Humility was negatively related to inspirational motivation and high performance expectations. Face and power-resources were negatively related to inspirational motivation, intellectual stimulation, and fostering acceptance of group goals behaviors. Power-dominance was negatively associated with intellectual stimulation and fostering acceptance of group goals. Finally, stimulation and self-direction action were positively related to intellectual stimulation, and stimulation was also positively associated with inspirational motivation. With regard to unreliable findings, coaches who emphasized basic values of tradition, conformity (rules and interpersonal), security (personal and societal), achievement, hedonism, and self-direction action were unrelated to any of the transformational behaviors, and no basic values were related to appropriate role model behaviors.

**Table 4:** Bivariate correlations between basic values and transformational leadership behaviors (*N* = 266 coaches).

	Transformational leadership behaviors					
	IC	IM	IS	FAG	HPE	ARM
Universalism-concern	0.17**	0.04	0.15**	0.18**	0.09	0.06
Universalism-nature	0.10	0.09	0.21**	0.10	-0.01	-0.01
Universalism-tolerance	0.20**	0.11	0.20**	0.22**	0.04	0.04
Benevolence-dependability	0.05	0.05	0.07	0.15*	0.03	0.09
Benevolence-caring	0.02	0.09	0.03	0.20**	0.11	0.07
Tradition	-0.03	0.01	-0.10	0.04	0.07	0.08
Humility	-0.08	-0.14*	-0.09	-0.11	-0.15*	-0.02
Conformity-rules	0.06	0.01	0.04	0.07	-0.03	0.11
Conformity-interpersonal	-0.03	-0.12	-0.04	-0.11	-0.08	-0.05
Security-personal	-0.03	0.01	-0.04	0.07	-0.03	0.07
Security-societal	-0.07	-0.01	0.07	-0.02	0.01	0.01
Face	-0.09	-0.13*	-0.17**	-0.20**	-0.07	-0.10
Power-dominance	-0.10	-0.11	-0.18**	-0.24**	0.01	0.01
Power-resources	-0.11	-0.13*	-0.23**	-0.24**	-0.04	-0.06
Achievement	-0.09	0.10	-0.11	-0.11	0.09	-0.03
Hedonism	-0.03	0.02	0.01	0.04	-0.08	-0.11
Stimulation	0.10	0.17**	0.19**	0.11	0.09	-0.02
Self-direction-thought	0.07	0.11	0.13*	0.09	0.02	-0.05
Self-direction-action	0.02	-0.01	0.06	0.08	0.01	-0.05

*IC, individual consideration; IM, inspirational motivation; IS, intellectual stimulation; FAG, fostering acceptance of group goals; HPE, high performance expectations; ARM, appropriate role model. ^∗^p < 0.05, ^∗∗^p < 0.01, two-tailed. Score-centered responses for each basic value were used.*

### Moderation and Mediation Effect

Before testing the moderation and mediation effects, correlation analyses were conducted between the value dimensions, perceived club autonomy support and pressure, and transformational coach behaviors (see **Table 5**). Self-transcendence and openness to change values correlated positively with inspirational motivation, intellectual stimulation, and fostering acceptance of group goals behaviors. Conservation correlated negatively with inspirational motivation and intellectual stimulation; and self-enhancement correlated negatively with individual consideration, intellectual stimulation, and fostering acceptance of group goals. Self-transcendence also correlated positively with individual consideration. Moreover, self-transcendence was positively related to a perceived club autonomy supportive environment and negatively associated with perceived club pressure, whereas the contrary was observed in the relationship between self-enhancement and perceived club autonomy support, which was negative, whereas the relationship between self-enhancement and perception of pressure at the club was positive. Openness to change was negatively associated with the perception of pressure at the club.

**Table 5:** Bivariate correlations between value dimensions, transformational leadership behaviors, and perceived club autonomy and pressure (*N* = 266 coaches).

Variables	1	2	3	4	5	6	7	8	9	10	11
(1) Self-transcendence	–										
(2) Conservation	-0.43**	–									
(3) Self-enhancement	-0.58**	-0.25**	–								
(4) Openness to change	0.30**	-0.78**	-0.15**	–							
(5) Individual consideration	0.21**	-0.08	-0.13*	0.07	–						
(6) Inspirational motivation	0.15**	-0.12*	-0.08	0.12*	0.34**	–					
(7) Intellectual stimulation	0.27**	-0.10*	-0.24**	0.15**	0.43**	0.41**	–				
(8) Fostering goal acceptance	0.30**	-.08	-0.27**	0.12*	0.36**	0.46**	0.46**	–			
(9) High performance expectations	0.08	-0.08	0.01	0.03	0.24**	0.30**	0.25**	0.38**	–		
(10) Appropriate role model	0.08	0.03	-0.04	-0.08	0.33**	0.30**	0.31**	0.36**	0.33**	–	
(11) Perceived club autonomy	0.22**	-0.04	-0.21**	0.08	0.17**	0.18**	0.21**	0.25**	0.10*	0.18**	–
(12) Perceived club pressure	-0.27**	0.05	0.27**	-0.11*	-0.16**	-0.07	-0.09	-0.21**	-0.05	-0.20**	-0.47**

*^∗^p < 0.05, ^∗∗^p < 0.01, one-tailed. Centered responses for each value dimension were used.*

**FIGURE 3:**
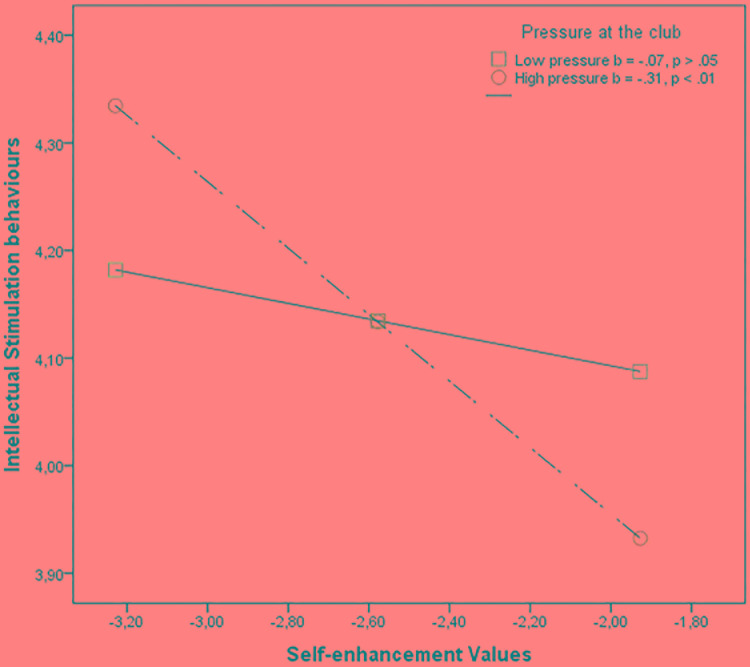
Associations between the self-enhancement values and the intellectual stimulation behaviors as a function of perception of pressure at the club.

To test whether perceptions of an autonomy supportive environment and perceptions of a pressure environment at the club would moderate the relationship between value dimensions and transformational behaviors, parameters for regression equations were estimated. Specifically, we examined perceptions of an autonomy supportive environment and perceptions of a pressure environment as moderators in the associations between each of the four value dimensions and each of the six transformational behaviors. Results showed that perceptions of an autonomy supportive environment did not moderate the relationship between value dimensions and transformational behaviors; however, the relationship between the self-enhancement value and intellectual stimulation was moderated by perceptions of a pressure environment (see **Table 6**). For coaches who hold self-enhancement values, high pressure at the club is a risk factor that significantly reduces the use of intellectual stimulation behaviors (see **Figure 3**).

**Table 6:** Results testing the moderation effects of perceived club autonomy and perceived club pressure in the relationship between value dimensions and transformational leadership behaviors (*N* = 266 coaches).

Outcomes Variables	IC	IM	IS	FAG	HPE	ARM
Self-transcendence	0.24**	0.16	0.32**	0.35**	0.03	-0.02
Moderator PCA	-0.01	-0.06	0.04	-0.03	-0.01	0.01
Moderator PCP	0.05	0.07	0.16	0.12	0.23	0.15
Conservation	-0.07	-0.17	-0.11	-0.09	-0.09	0.06
Moderator PCA	0.13	-0.03	0.09	0.05	0.12	0.05
Moderator PCP	0.04	0.07	0.06	-0.05	-0.01	-0.14
Self-enhancement	-0.08	-0.04	-0.18**	-0.18**	0.03	0.02
Moderator PCA	-0.03	0.06	-0.05	0.01	-0.02	0.02
Moderator PCP	-0.02	-0.07	-0.17**	-0.05	-0.08	0.02
Openness to change	0.02	0.12	0.13	0.09	-0.02	-0.14*
Moderator PCA	-0.14	-0.04	-0.10	-0.06	-0.16	-0.12
Moderator PCP	-0.16	-0.11	-0.09	-0.09	-0.13	0.01

*PCA, perceived club autonomy; PCP, perceived club pressure; IC, individual consideration; IM, inspirational motivation; IS, intellectual stimulation; FAG, fostering acceptance of group goals; HPE, high performance expectations; ARM, appropriate role model. Score-centered responses for each value dimension were used. ^∗∗^p < 0.01, ^∗^p < 0.05*.

**Table 7** presents the results of the regression analyses testing whether perceptions of an autonomy supportive environment and perceptions of a pressure environment would mediate the relationship between each of the four value dimensions and each of the six transformational behaviors. Results revealed a positive and significant association between self-transcendence and perceptions of an autonomy supportive environment, and a negative and significant association with perceptions of a pressure environment. Holding a self-transcendence value directly and positively predicted individual consideration, inspirational motivation, intellectual stimulation, and fostering acceptance of group goals behaviors. Indeed, holding a self-transcendence value indirectly and positively predicted inspirational motivation, intellectual stimulation, and fostering acceptance of group goals behaviors through its effect on perceptions of an autonomy supportive (partial mediation). Moreover, holding a self-transcendence value indirectly and positively predicted appropriate role model through its effect on perceptions of a pressure environment (total mediation). The conservation value directly and negatively predicted inspirational motivation. Self-enhancement directly and negatively predicted intellectual stimulation and fostering acceptance of group goals, and it also indirectly and negatively predicted intellectual stimulation and fostering acceptance of group goals via its effect on perceptions of an autonomy supportive (partial mediation). Self-enhancement indirectly and negatively predicted inspirational motivation and appropriate role model through its effect on perceptions of an autonomy supportive environment and on perceptions of a pressure environment, respectively (total mediation). Finally, the openness to change value directly and positively predicted inspirational motivation and intellectual stimulation behaviors. All the models tested predicted a significant percentage of the variance in each transformational behavior, with the exception of high performance expectations, which was not significant (see **Table 7**).

**Table 7:** Results testing the mediation effects of perceived club autonomy and perceived club pressure between value dimensions and transformational leadership behaviors (*N* = 266 coaches).

Models	PCA	PCP	IC	IC		IM	IM		I	IS	
	
			DE	IE	*R*^2^ IC	DE	IE	*R*^2^ IM	DE	IE	*R*^2^ IS
**Self-transcendence**	0.69**	-0.71**	0.25**		0.05**	0.20*		0.02**	0.36**		0.07**
Mediator PCA	–	–	0.05	0.03		0.09*	0.06sig		0.08**	0.06sig	
Mediator PCP	–	–	-0.03	0.02		0.03	-0.02		0.03	-0.02	
*R*^2^ total model	–	–			0.06**			0.05**			0.10**
**Conservation**	-0.12	0.15	-0.10		0.01	-0.17*		0.01*	-0.13		0.01
Mediator PCA	–	–	0.06*	-0.01		0.09**	-0.01		0.10**	-0.01	
Mediator PCP	–	–	-0.05	-0.01		0.01	0.00		0.01	0.00	
*R*^2^ total model	–	–			0.04*			0.04**			0.05**
**Self-enhancement**	-0.36**	0.38**	-0.07		0.02*	-0.04		0.01	-0.17**		0.06**
Mediator PCA	–	–	0.05*	-0.02		0.09**	-0.03sig		0.08**	-0.03sig	
Mediator PCP	–	–	-0.04	-0.01		0.02	0.01		0.03	0.01	
*R*^2^ total model	–	–			0.05**			0.03*			0.08**
**Openness to change**	0.19	-0.24*	0.06		0.00	0.13*		0.01*	0.16*		0.02*
Mediator PCA	–	–	0.06*	0.01		0.09**	0.02		0.09**	0.02	
Mediator PCP	–	–	-0.05	0.01		0.02	-0.00		0.01	-0.00	
*R*^2^ total model	–	–			0.04*			0.04**			0.06**

**Models**	**PCA**	**PCP**	**FAG**	**FAG**		**HPE**	**HPE**		**ARM**	**ARM**	
	
			**DE**	**IE**	***R*^2^ FAG**	**DE**	**IE**	***R*^2^ HPE**	**DE**	**IE**	***R*^2^ ARM**

**Self-transcendence**	0.69**	-0.71**	0.39**		0.09**	0.10		0.01	0.02		0.01
Mediator PCA	–	–	0.08*	0.06sig		0.05	0.03		0.05	0.03	
Mediator PCP	–	–	-0.03	0.02		0.01	-0.00		-0.07*	0.05sig	
*R*^2^ total model	–	–			0.13**			0.02			0.05**
**Conservation**	-0.12	0.15	-0.10		0.00	-0.11		0.01	0.06		0.00
Mediator PCA	–	–	0.09**	-0.01		0.05	-0.01		0.05	-0.01	
Mediator PCP	–	–	-0.07*	-0.01		0.00	0.00		-0.08*	-0.01	
*R*^2^ total model	–	–			0.08**			0.02			0.05**
**Self-enhancement**	-0.36**	0.38**	-0.18**		0.07**	0.04		0.00	0.02		0.00
Mediator PCA	–	–	0.08**	-0.03sig		0.05	-0.02		0.05	-0.02	
Mediator PCP	–	–	-0.04	-0.01		-0.01	-0.00		-0.08*	-0.03sig	
*R*^2^ total model	–	–			0.12**			0.02			0.05**
**Openness to change**	0.19	-0.24*	0.11		0.01*	0.02		0.00	-0.11		0.01
Mediator PCA	–	–	0.09**	0.02		0.05			0.05	0.01	
Mediator PCP	–	–	-0.06	0.01		-0.00			-0.08*	0.02	
*R*^2^ total model	–	–			0.08**			0.01			0.06**

*PCA, perceived club autonomy; PCP, perceived club pressure; IC, individual consideration; IM, inspirational motivation; IS, intellectual stimulation; FAG, fostering acceptance of group goals; HPE, high performance expectations; ARM, appropriate role model; DE, direct effect; IE, indirect effect. Score-centered responses for each value dimension were used. ^∗∗^p < 0.01 and ^∗^p < 0.05, sig, significant indirect effect.*

## Discussion

A large amount of literature has examined values and their relationship with behaviors (e.g., Maio et al., 2009; Roccas and Sagiv, 2017). Nevertheless, no previous study has assessed the relationship between the refined values and transformational leaders’ behaviors. Using the refined theory of basic individual values (Schwartz et al., 2012) and transformational leadership theory as frameworks (Bass, 1985), this study explored the associations between basketball coaches’ value priorities and their transformational leadership behaviors, testing the potential mediation versus moderation effect of two alternative variables in this relationship: perceived club pressure and an autonomy supportive environment.

As expected, and consistent with previous research in the organizational context (e.g., Kanungo and Mendonca, 1996; Bass and Steidlmeier, 1999; Sarros and Santora, 2001; Sosik, 2005), the study results showed that the stronger the intensity of the coach’s self-transcendent values (i.e., universalism and benevolence), the more likely the coach is to display transformational behaviors and perceive a more autonomy supportive environment and lower pressure from the club. However, coaches who held beliefs in self-enhancement values (i.e., power) displayed lower transformational behaviors and perceived higher pressure from the club and a less autonomy supportive environment.

Specifically, coaches who express concern (universalism-concern), tolerance (universalism-tolerance), and care (benevolence-caring) for others and are reliable and trustworthy members of the in-group display these values by considering the players’ individual needs (individual consideration), empowering their players by thinking in different ways (intellectual stimulation), asking them to do their best (inspirational motivation), and creating team spirit (fostering acceptance of group goals). Moreover, coaches who consider excitement, novelty, challenge (stimulation), creativity, curiosity, and interest (self-direction thought) to be important values express them by communicating an optimistic and realistic vision of future team achievements (inspirational motivation) and reflecting each team member’s goal, as well as sharing how to reach their goals with the team (intellectual stimulation). Our findings are consistent with previous researchers (e.g., Kanungo and Mendonca, 1996; Bass and Steidlmeier, 1999; Sarros and Santora, 2001; Sosik, 2005) who found that self-transcendence values (i.e., universalism, benevolence, and altruism) support transformational leadership behaviors. Indeed, Sosik (2005) also identified openness to change values (i.e., stimulation and self-direction) associated with these behaviors.

Our results indicate that coaches attributed greater importance to self-transcendence and openness to change values, and this is reflected through transformational behaviors. In model (Schwartz, 1992; Schwartz et al., 2012) some value types complemented each other, whereas others were in conflict. Accordingly, the bipolar dimension of *conservation* versus *self-enhancement* may have a negative relationship or no relationship with transformational leadership behaviors. Our findings confirm this suggestion and show that coaches who are self-effacing rather than boastful (humility) and want to maintain their public image and avoid humiliation (face) (both conservation values) will avoid generating visions of future group/team states (inspirational motivation). Humility consists of accepting life’s circumstances without expecting more, thus maintaining their public image, which is the opposite of what inspirational motivation means. Furthermore, coaches who want to promote themselves by controlling athletes (power dominance) and material resources (power resources) (both self-enhancement values) do not empower their athletes through thinking in different ways (intellectual stimulation) or foster the acceptance of team goals (fostering acceptance of group goals) because these coaches are interested in their own personal goals.

Our results are consistent with theory of human values Schwartz (1992); however, some researchers have presented contradictory or incongruent results for values and behavior. For example, Sarros and Santora (2001) found a positive correlation between self-enhancement and transformational behaviors. In the same vein, Sosik (2005) reported that self-transcendence and self-enhancement were both positively related to charismatic leadership (i.e., inspirational stimulation and appropriate role model). A possible explanation could be the difference established in previous research between authentic and unauthentic transformational leaders (Hogan et al., 1990; Kanungo and Mendonca, 1996; Bass and Steidlmeier, 1999). Bass and Steidlmeier (1999) used leader values and morals to explain the differences between transformational and pseudo-transformational leaders. For instance, transformational leaders feel responsible for and concerned about the group’s achievement, whereas pseudo-transformational leaders are more concerned about their individual achievements. With regard to the latter, Bass and Steidlmeier stated: ‘They are captains who sail under false colors. They are spiritual leaders who are false prophets’ (p. 188). Consequently, according to Bass and Steidlmeier (1999), leaders who display transformational behaviors but hold self-enhancement and conservation values may be considered pseudo-transformational leaders. Sometimes people can present behavior that is contrary to their values in order to conform to the group. To prevent inconsistency and avoid problems when comparing values of different individuals or correlating values with other variables, the results should be compared with those from studies that use the same instruments, and according to Schwartz (1992, 2006), respondents’ ratings should be centered. Otherwise, the results can be different (none of the previous studies specified centered their score values).

Self-transcendence values (universalism and benevolence) are opposed to self-enhancement values (power and achievement). Power and achievement values emphasize authority and control over people and resources, as well as personal success beyond the group, whereas universalism and benevolence emphasize concern for the welfare of others. Our results suggest that some transformational behaviors reflect values of self-transcendence (individual consideration, inspirational motivation, intellectual stimulation, and fostering acceptance of group goals) and openness to change (inspirational motivation and intellectual stimulation), whereas some transformational behaviors are shown to be opposed to self-enhancement (intellectual stimulation and fostering acceptance of group goals) and conservation (inspirational motivation) values. Therefore, and in terms of Bardi and Schwartz (2003), intellectual stimulation and fostering acceptance of group goals may be considered *value-expressive behaviors of* self-transcendence, and inspirational motivation can be considered a *value-expressive behavior* of openness to change because these behaviors reflect values that are incompatible with the opposing values in the circle.

In terms of the perceptions of the club environment, our findings show that when coaches hold the welfare of others as a more important life value, they are more likely to perceive higher autonomy support at the club and lower pressure; whereas when coaches consider social status and prestige or control and dominance over other people to be important values, they are more likely to perceive less autonomy support and higher pressure at the club. However, in general, these club perceptions do not affect the expression of values, although they can enhance or diminish the relationship. That is, when coaches perceive an autonomy supportive environment at the club (i.e., a favorable context), self-transcendence values are more likely to be expressed through inspirational motivation, intellectual stimulation, and fostering acceptance of group goals behaviors, whereas the perception of pressure at the club (i.e., an unfavorable context) does not decrease the likelihood that these values will be expressed through these transformational behaviors. On the other hand, perceiving a high autonomy supportive environment cushioned the negative associations between self-enhancement and intellectual stimulation and fostering acceptance of group goals. However, coaches with self-enhancement values tend to avoid intellectual stimulation to a greater extent when they perceive high levels of pressure at the club.

Our findings confirm previous suggestions (e.g., Bardi and Schwartz, 2003) that values can influence behavior more or less due to the social context (autonomy supportive versus pressure environments). When situational pressures are non-existent, people have a greater chance of reporting that their behavior is consistent with their values. Nevertheless, under heavy pressure rules, values can be opposite to behaviors when people want to conform to the group. Perhaps for this reason, in the case of self-enhancement values, behaviors related to inspirational motivation receive a more negative and significant contribution of the variance related to perception of an autonomy supportive environment than the corresponding value itself. Although several studies have explained the association between values and behaviors, few studies have examined the mediators in this association. In general, the results of this study showed that the relationship between values and transformational behaviors was partially mediated by the perception of an autonomy supportive environment provided by the club. However, this relationship was not mediated by the perception of pressure at the club. Further studies are needed to investigate other potential mediators, in addition to perceptions of autonomy supportive or pressure environments, when examining the associations between personal values and the use of transformational behaviors. For example, as point out by Schwartz (2017), the correlations between values and specific behaviors are rarely very strong because attitudes mediate relationships between values and behavior in most instances. Moreover, in order for a value to exert influence, it must be activated, and in a particular context important values can be activated more often and have more influence.

The findings from our study extend previous findings on the association between values and behaviors by suggesting that this association is also evident in basketball coaches. Coaches who hold values that promote the welfare and acceptance of close and distant others as equals display more transformational behaviors. By contrast, coaches who endorse or give priority to self-enhancement values such as power dominance and power resources are likely to display fewer transformational behaviors.

Strengths of our study include the use of a large sample of basketball coaches. Additionally, the assessment of basic values and transformational leadership behaviors provides detailed information about specific behaviors and the way values and these behaviors fit together. No other studies were found on the associations among coaches’ values, perceptions of autonomy supportive and pressure environments at the club, and transformational coach behaviors.

Some limitations need to be considered when interpreting the results. We cannot establish any causal relationships based on our data due to the cross-sectional design. Instead, the results have to be viewed as evidence of potential pathways that require longitudinal investigation. The sample was unbalanced in terms of gender and the sport(s) coached because it was limited to basketball and females were under-represented. The lack of female coaches is common in our country, and so it is representative of our reality. Further research is needed to extend the generalizability of these findings to other sports. The alpha coefficients for some scales were problematic to some extent; however, hypothesized relationships that were significant emerged in the expected direction. Another limitation is related to the use of self-report measures, which are very common in sport psychology research. Future studies would benefit from the use of objective variables, such as observational methods to evaluate coaches’ behaviors or even autonomy supportive versus pressure environments provided at the club. Despite these limitations, the results of this study contribute to the literature by providing information about the link between values and behaviors using a sample of basketball coaches, thus extending previous research suggesting that values can be considered a major influence on behavior in the sport context.

### Practical Implications

In light of the results of this study, we suggest some practical implications for sport consultants. Although transformational leadership behaviors have been established as being very effective in achieving better results in sports, it is very important to know which values are important for the coaches we are working with, as well as their perceptions of the club environments. Thus, coaches who have a high level of self-enhancement and conservation values may be reluctant to display some transformational behaviors. This resistance will increase if they perceive high levels of pressure and low levels of autonomy support at their clubs. For instance, intellectual stimulation has been suggested as a highly effective leadership behavior; however, coaches who hold high self-enhancement values may not believe in the advantage of using this behavior. Therefore, if the sport psychologist is not aware of this situation, the success of the coaching consultancy could be compromised.

## Author Contributions

All authors carried out the design and drafted the article. IC was responsible for ethical approval. FA was responsible for collecting the data. IC and OA conducted statistical analyses. All authors read and approved the final version of the article and agree with the order of presentation of the authors.

## Conflict of Interest Statement

The authors declare that the research was conducted in the absence of any commercial or financial relationships that could be construed as a potential conflict of interest.

## Footnotes

1 http://www.psych.rochester.edu/SDT/
