# Supplementary material for: Relationships Between Personal Values and Leadership Behaviors in Basketball Coaches
Source: Front Psychol. 2018 Sep 12;9:1661. doi: 10.3389/fpsyg.2018.01661 (PMC6143767; doi:10.3389/fpsyg.2018.01661)
Supplement: Supplementary file 1 [file Table_1.DOCX]

**RESULTS OF THE CONFIRMATORY FACTOR ANALYSES OF THE INSTRUMENTS**

**1. COACH VALUES: PORTRAIT VALUES QUESTIONNAIRE (Schwartz et al., 2012)**

**57 items 🡪 19 basic values 🡪 value dimensions: self-transcendence, conservation, self-enhancement and openness to change**

**CFA 🡪 SELF-TRANSCENDENCE VALUES**

LAMBDA-X

UNIVNAT UNIVPRE UNIVTO BENECUI BENEDEP

-------- -------- -------- -------- --------

PVQR5 - - 0.601 - - - - - -

(0.059)

10.157

PVQR8 0.887 - - - - - - - -

(0.050)

17.711

PVQR11 - - - - - - 0.715 - -

(0.059)

12.168

PVQR14 - - - - 0.647 - - - -

(0.060)

10.715

PVQR19 - - - - - - - - 0.614

(0.059)

10.316

PVQR21 0.822 - - - - - - - -

(0.052)

15.833

PVQR25 - - - - - - 0.797 - -

(0.057)

13.938

PVQR27 - - - - - - - - 0.794

(0.055)

14.431

PVQR34 - - - - 0.740 - - - -

(0.058)

12.660

PVQR37 - - 0.833 - - - - - -

(0.053)

15.656

PVQR45 0.886 - - - - - - - -

(0.050)

17.698

PVQR47 - - - - - - 0.645 - -

(0.060)

10.705

PVQR52 - - 0.841 - - - - - -

(0.053)

15.863

PVQR55 - - - - - - - - 0.830

(0.054)

15.305

PVQR57 - - - - 0.700 - - - -

(0.059)

11.820

**CFA 🡪 CONSERVATION VALUES**

LAMBDA-X

SEGPER SEGSOC TRADI CONFREGL CONFINTE HUMIL FACE

-------- -------- -------- -------- -------- -------- --------

PVQR2 - - 0.646 - - - - - - - - - -

(0.059)

10.906

PVQR4 - - - - - - - - 0.650 - - - -

(0.059)

11.055

PVQR7 - - - - - - - - - - 0.363 - -

(0.074)

4.896

PVQR9 - - - - - - - - - - - - 0.754

(0.060)

12.539

PVQR13 0.523 - - - - - - - - - - - -

(0.064)

8.222

PVQR15 - - - - - - 0.789 - - - - - -

(0.055)

14.268

PVQR18 - - - - 0.746 - - - - - - - -

(0.058)

12.800

PVQR22 - - - - - - - - 0.759 - - - -

(0.056)

13.499

PVQR24 - - - - - - - - - - - - 0.449

(0.065)

6.891

PVQR26 0.677 - - - - - - - - - - - -

(0.061)

11.078

PVQR31 - - - - - - 0.769 - - - - - -

(0.056)

13.780

PVQR33 - - - - 0.785 - - - - - - - -

(0.058)

13.639

PVQR35 - - 0.685 - - - - - - - - - -

(0.059)

11.710

PVQR38 - - - - - - - - - - 0.674 - -

(0.079)

8.496

PVQR40 - - - - 0.744 - - - - - - - -

(0.058)

12.756

PVQR42 - - - - - - 0.805 - - - - - -

(0.055)

14.666

PVQR49 - - - - - - - - - - - - 0.782

(0.060)

13.063

PVQR50 - - 0.897 - - - - - - - - - -

(0.055)

16.319

PVQR51 - - - - - - - - 0.862 - - - -

(0.054)

15.957

PVQR53 0.669 - - - - - - - - - - - -

(0.061)

10.935

PVQR54 - - - - - - - - - - 0.458 - -

(0.074)

6.232

**CFA 🡪 SELF-ENHANCEMENT VALUES**

LAMBDA-X

ACHIEV POWERDO POWERRE

-------- -------- --------

PVQR6 - - 0.601 - -

(0.062)

9.764

PVQR12 - - - - 0.696

(0.059)

11.890

PVQR17 0.310 - - - -

(0.068)

4.573

PVQR20 - - - - 0.872

(0.055)

15.742

PVQR29 - - 0.787 - -

(0.059)

13.278

PVQR32 0.830 - - - -

(0.066)

12.565

PVQR41 - - 0.812 - -

(0.059)

13.767

PVQR44 - - - - 0.665

(0.059)

11.238

PVQR48 0.627 - - - -

(0.065)

9.688

**CFA 🡪 OPENNESS TO CHANGE VALUES**

LAMBDA-X

SELFPEN SELFACC STIMULA HEDONIS

-------- -------- -------- --------

PVQR1 0.454 - - - - - -

(0.063)

7.216

PVQR3 - - - - - - 0.505

(0.062)

8.205

PVQR10 - - - - 0.600 - -

(0.059)

10.238

PVQR16 - - 0.806 - - - -

(0.056)

14.348

PVQR23 0.712 - - - - - -

(0.058)

12.220

PVQR28 - - - - 0.731 - -

(0.056)

13.083

PVQR30 - - 0.449 - - - -

(0.063)

7.131

PVQR36 - - - - - - 0.824

(0.054)

15.169

PVQR39 0.720 - - - - - -

(0.058)

12.364

PVQR43 - - - - 0.932 - -

(0.051)

18.137

PVQR46 - - - - - - 0.844

(0.054)

15.665

PVQR56 - - 0.686 - - - -

(0.058)

11.792

**2. PERCEIVED CLUB AUTONOMY: SPORT CLIMATE QUESTIONNAIRE (Balaguer et al., 2009; http://www.psych.rochester.edu/SDT/)**

**CFA 🡪 PERCEIVED CLUB AUTONOMY SUPPORT** **QUESTIONNAIRE**

LAMBDA-X

autonomy

--------

AA1 0.568

(0.060)

9.407

AA2 0.551

(0.061)

9.078

AA3 0.813

(0.054)

15.083

AA4 0.688

(0.057)

11.979

AA5 0.782

(0.055)

14.266

AA6 0.670

(0.058)

11.552

AA7 0.618

(0.059)

10.430

**3. PERCEIVED CLUB PRESSURE: CONSTRAINTS AT WORK SCALE (Pelletier et al., 2002)**

**CFA 🡪 PERCEIVED CLUB PRESSURE** **QUESTIONNAIRE**

LAMBDA-X

pressure

--------

PRES1 0.825

(0.053)

15.422

PRES2 0.613

(0.059)

10.372

PRES3 0.598

(0.059)

10.051

PRES4 0.743

(0.056)

13.327

PRES5 0.854

(0.053)

16.206

**4. COACH BEHAVIORS: DIFFERENTIATED TRANSFORMATIONAL LEADERSHIP INVENTORY (Callow et al., 2009; Vella et al., 2012)**

**23 items 🡪 6 behaviors 🡪 individual consideration, inspirational motivation, intellectual stimulation, fostering acceptance of group goals and promoting teamwork, high performance expectations, and appropriate role model**

**CFA 🡪 TRANSFORMATIONAL LEADERSHIP** **INVENTORY**

LAMBDA-X

consi moti estimu goals expecta role

-------- -------- -------- -------- -------- --------

LID1 - - - - 0.441 - - - - - -

(0.064)

6.911

LID2 0.337 - - - - - - - - - -

(0.067)

5.064

LID3 - - 0.634 - - - - - - - -

(0.060)

10.571

LID4 0.764 - - - - - - - - - -

(0.062)

12.337

LID5 - - 0.700 - - - - - - - -

(0.058)

12.002

LID6 - - 0.575 - - - - - - - -

(0.061)

9.375

LID7 - - - - 0.640 - - - - - -

(0.060)

10.701

LID8 - - - - 0.760 - - - - - -

(0.057)

13.368

LID9 0.331 - - - - - - - - - -

(0.067)

4.967

LID10 - - - - - - 0.740 - - - -

(0.057)

13.001

LID11 - - - - - - - - 0.651 - -

(0.065)

10.090

LID12 - - - - - - 0.832 - - - -

(0.055)

15.241

LID13 0.538 - - - - - - - - - -

(0.064)

8.455

LID14 - - - - - - - - - - 0.718

(0.069)

10.364

LID15 - - - - - - - - 0.680 - -

(0.064)

10.596

LID16 - - 0.856 - - - - - - - -

(0.055)

15.683

LID17 - - - - - - - - - - 0.322

(0.072)

4.490

LID18 - - - - 0.846 - - - - - -

(0.055)

15.452

LID19 - - - - - - - - 0.452 - -

(0.068)

6.676

LID20 - - - - - - 0.704 - - - -

(0.058)

12.167

LID21 - - - - - - - - - - 0.387

(0.071)

5.451

LID22 - - - - - - - - - - 0.599

(0.069)

8.709

LID23 - - - - - - - - 0.688 - -

(0.064)

10.742
