# Supplementary material for: Relationships Between Personal Values and Leadership Behaviors in Basketball Coaches
Source: Front Psychol. 2018 Sep 12;9:1661. doi: 10.3389/fpsyg.2018.01661 (PMC6143767; doi:10.3389/fpsyg.2018.01661)
Supplement: Supplementary file 2 [file Table_2.DOCX]

Table 4b

*Bivariate correlations between basic values and transformational leadership behaviours (N = 266 coaches)*

|  | Transformational leadership behaviours | | | | | | | | | | | |  |
| --- | --- | --- | --- | --- | --- | --- | --- | --- | --- | --- | --- | --- | --- |
|  | Individual consideration | | Inspirational motivation | | Intellectual stimulation | | Fostering acceptance of group goals | | High performance expectations | | Appropriate role model | |  |
| Universalism-concern | | .32** | | .20** | | .31** | | .38** | | .22** | | .23** | |
| Universalism-nature | | .21** | | .19** | | .31** | | .24** | | .09 | | .11 | |
| Universalism-tolerance | | .36** | | .26** | | .37** | | .42** | | .17** | | .21** | |
| Benevolence-dependability | | .26** | | .24** | | .29** | | .40** | | .20** | | .29** | |
| Benevolence-caring | | .24** | | .29** | | .26** | | .45** | | .27** | | .27** | |
| Tradition | | .09 | | .11 | | .04 | | .18** | | .15* | | .19** | |
| Humility | | .09 | | .02 | | .09 | | .10 | | -.01 | | .15* | |
| Conformity-rules | | .18** | | .13* | | .18** | | .23** | | .08 | | .22** | |
| Conformity-interpersonal | | .10 | | .02 | | .11 | | .06 | | .03 | | .08 | |
| Security-personal | | .15* | | .16** | | .15* | | .27** | | .11 | | .22** | |
| Security-social | | .07 | | .11 | | .20** | | .15* | | .11 | | .13* | |
| Face | | .06 | | .01 | | -.01 | | .01 | | .04 | | .05 | |
| Power-dominance | | .04 | | .02 | | -.03 | | -.06 | | .10 | | .13* | |
| Power-resources | | .03 | | .01 | | -.08 | | -.06 | | .08 | | .08 | |
| Achievement | | .09 | | .25** | | .08 | | .11 | | .21** | | .14* | |
| Hedonism | | .17** | | .20** | | .22** | | .29** | | .08 | | .10 | |
| Stimulation | | .23** | | .28** | | .31** | | .27** | | .19** | | .12 | |
| Self-direction-thought | | .29** | | .30** | | .36** | | .36** | | .19** | | .16** | |
| Self-direction-action | | .22** | | .18** | | .26** | | .32** | | .16** | | .15* | |

*Note*. Not centred responses for each value dimension were used. **p* < .05; ***p* < .01, one-tailed.

Table 5b

*Bivariate correlations between the four value dimensions, transformational leadership behaviours, and perceived club autonomy and pressure (N = 266 coaches)*

| Variables | 1 | 2 | 3 | 4 | 5 | 6 | 7 | 8 | 9 | 10 | 11 |
| --- | --- | --- | --- | --- | --- | --- | --- | --- | --- | --- | --- |
| 1. Self-transcendence | - |  |  |  |  |  |  |  |  |  |  |
| 2. Conservation | .43** | - |  |  |  |  |  |  |  |  |  |
| 3. Self-enhancement | -.03 | .22** | - |  |  |  |  |  |  |  |  |
| 4. Openness to change | .59** | .07 | .10 | - |  |  |  |  |  |  |  |
| 5. Individual Consideration | .39** | .16** | .07 | .31** | - |  |  |  |  |  |  |
| 6. Inspirational Motivation | .33** | .12* | .10 | .33** | .34** | - |  |  |  |  |  |
| 7. Intellectual Stimulation | .44** | .16** | -.02 | .39** | .43** | .41** | - |  |  |  |  |
| 8. Fostering goal acceptance | .52** | .22** | -.01 | .42** | .36** | .46** | .46** | - |  |  |  |
| 9. High performance expectations | .25** | .12 | .16** | .22** | .24** | .30** | .25** | .38** | - |  |  |
| 10. Appropriate role model | .30** | .23** | .15* | .18** | .33** | .30** | .31** | .36** | .33** | - |  |
| 11. Perceived club autonomy | .30** | .11 | -.08 | .21** | .17** | .18** | .21** | .25** | .10 | .18** | - |
| 12. Perceived club pressure | -.20** | .01 | .23** | -.11 | -.16* | -.07 | -.09 | -.21** | -.05 | -.20** | -.47** |

*Note*. Not centred responses for each value dimension were used. **p* < .05; ***p* < .01, one-tailed.
